# Cost-effectiveness analysis of a school-based dental caries prevention program using fluoridated milk in Bangkok, Thailand

**DOI:** 10.1186/s12903-018-0485-7

**Published:** 2018-02-15

**Authors:** Rodrigo Mariño, Fernando Traub, Puangtong Lekfuangfu, Kornkamol Niyomsilp

**Affiliations:** 1 Melbourne Dental School, Oral Health Cooperative Research Centre, University of Melbourne, Melbourne, VIC 3010 Australia; 2 Dental Health Bureau, Department of Health, Ministry of Public Health, Bangkok, Thailand

**Keywords:** Dental caries, Fluoridation, Thailand

## Abstract

**Background:**

This study modelled the cost-effectiveness, from a societal perspective, of a program that used fluoridated milk to prevent dental caries in children who were 6 years old at the beginning of the program, versus non-intervention, after 6 years.

**Methods:**

After 6 years, children in the milk-fluoridation program had a significant (34%) reduction in dental caries experience compared to those in the comparison community (i.e., received school milk without added fluoride) (DMFS: 1.06 vs. 1.60).

**Results:**

This improvement was achieved with an investment of Thailand Baht (THB) 5,345,048 over 6 years (or THB 11.88 per child, per year) (1 US$ = THB(2011) 30.0). When comparing the costs of the operation of the program and dental treatment in the test community with those of the comparison community, the program resulted in a net societal savings of THB 8,177,179 (range 18,597,122 to THB 7,920,711) after 6 years. This investment would result in 40,500 DMFS avoided in a community with a childhood population of 75,000 [DMFS avoided: 75,000 x (− 0.54)].

**Conclusions:**

While the analysis has inherent limitations due to its dependence on a range of assumptions, the results suggest that, from a societal perspective, when compared with the non-intervention group, the Bangkok Metropolitan Administration intervention appeared to be a more cost-efficient option than current standard oral health care.

## Background

The use of fluorides is recognized as one of the most successful measures for the prevention of disease in the history of public health [1]. Fluoride can be delivered to individuals as a dental preventive measure through a variety of mechanisms. The most commonly used vehicles for community-based fluoridation programs are drinking water and salt fluoridation. Other mechanisms include school water fluoridation, fluoride mouthrinses, topical application of fluoride solution gels and milk fluoridation [2–5].

In Thailand, the oral health promoting school program is well established. The program consists of oral health education and health promotion activities, regular oral health checkups, pit and fissure sealant programs and fluoride varnish [6]. The program also includes adequate fluoride exposure through supervised tooth brushing using fluoride toothpaste and in some schools, consumption of fluoridated milk. The first milk-fluoridation scheme was launched in Bangkok in 2000 and this has now been extended to eleven provinces, out of 77, involving more than one million children aged 3 to 12 years. Every child under this scheme receives, at no charge, 200 ml of fresh pasteurized milk per day (on school days) containing 0.5 mg F (sodium fluoride). Additionally, on holidays (i.e., 60 days), children receive containers of fluoridated UHT milk. Milk distribution is the responsibility of the local authority (e.g., Bangkok Metropolitan Administration [BMA]). Fluoridated milk is prepared by 20 dairy companies contracted to the local authority (e.g., BMA).

In the last few years, economic evaluations (EE) have become increasingly important in decision-making in health. Despite this, apart from EE of water fluoridation and dental sealants, the use and application of EE in dentistry remains limited [7, 8]. Furthermore, except for studies carried out on fluoridated milk programs in Chile [9, 10], there have been no true economic evaluations of dental caries prevention programs using milk as the vehicle, which reflect Thailand’s conditions.

The purpose of this evaluation was to assess the cost-effectiveness, from a societal viewpoint, of a dental caries prevention program using fluoridated milk in the city of Bangkok, Thailand, for children from 6 to 12 years old, versus non-intervention. The analysis calculated the per-unit and annual costs of the milk-fluoridation program under Thai conditions, and the cost of this program per child.

## Methods

The form of economic evaluation used in this study was cost-effectiveness analysis (CEA). In CEA, costs of alternative programs are measured as economic costs, and outcomes are measured in units of effectiveness [11]. This study used the “model” format for CEA [12]. This is an aggregate model with no interactions [13]. A decision tree examines populations undergoing different events (arms of the tree) and their associated costs and consequences [12]. Modelling was based, as far as possible, on real data and real values. When this was not possible, we used realistic assumptions based on secondary analysis of data collected as part of the regular practice of existing milk-fluoridation schemes operating in Thailand.

All costs were assessed from a societal perspective, using a 6-year time horizon. A societal viewpoint requires measurement of all costs and benefits to the community, no matter to whom they accrue [14, 15]. The Consolidated Health Economic Evolution Reporting Standards guideline was followed for reporting this economic evaluation [16].

### Cost

Cost for the fluoridated community were grouped into eight main categories:

1. Initial investment including the equipment required for implementation of the program and for quality control purposes. Calculations were done based on the assumptions that:
  - the same laboratory equipment necessary for sample analysis would be used in both pasteurized fluoridated-milk and UHT fluoridated-milk.
  - no equipment would be replaced during this 6-year period.
  - the equipment at the Royal Chitralada Project dairy^1^ would have the capacity to produce 16,500,200 ml bags of pasteurized milk per day and 75,000 cartons per day of UHT milk on every holiday. Thus, five plants would be required to produce 75,000 bags a day on school days, plus one plant for the 75,000 cartons per day of UHT milk.
2. Management of the program:
  - The program required a coordinator in charge of promotion and awareness in schools where the program is implemented. It was assumed that this role would require 4 h a week, with a wage of Thailand Bahts **(**THB**)** 87,000 a month for a full-time position in the 2011 financial year. The coordinator would require an administrative assistant (AA) with a monthly wage of THB 28,800.
  - The coordinator would prepare and submit a report and presentation to Her Royal Highness the Princess of Thailand every year. This would cost THB 10,000. This cost would be shared by six milk-fluoridation schemes operating in Thailand (i.e., THB 1667 per plant).
  - An office room for 4 h a week. The average office rent in the central business district of Bangkok is THB 750 per square meter per month [17]. It was assumed that a 9-square meter space would be necessary for normal operation of the program. Additionally, it was estimated that services including electricity, water, and internet would correspond to 20% of rent [18].
  3) Production costs: this included production costs and the cost of the analysis during the production process. Based on data from the Royal Chitralada Project, the cost per bag of pasteurized fluoridated-milk would be THB 0.00556, and the cost per carton of UHT fluoridated-milk would be THB 0.00678.
  During the production process, random samples would be taken to check Sodium Fluoride (NaF) concentration. Data from the Royal Chitralada Project indicated that the cost for each sample was THB 7.37. From the total daily production, sixteen 200 ml bags and 24 UHT cartons were tested for concentration levels. Additionally, there was a cost for instrument calibration of THB 97.92/day.
  Fluoridated-milk quality control analysis and monitoring: to check for the consistent provision of appropriate concentrations of NaF in the fluoridated-milk products, these samples were taken from schools to the Department of Health (DOH), Ministry of Public Health, with a cost of THB 200 per trip. Six samples of fluoridated-milk would be analysed from each of the dairy plants, eight times a year. In addition, the cost of the fluoride analysis at DOH would be THB 30 per sample.
  The cost of laboratory personnel technicians for samples handling and analysis was calculated assuming a monthly wage of THB 24,000 (THB 138.46/h) for a full-time equivalent position. It was considered that 66 h-a-year would be required to conduct the analyses.
  Additionally, the fluoride concentration in the milk was also monitored by determining fluoride urinary excretion. Information from the DOH indicated that the cost of the urinary analysis was THB 32,800. This cost was shared by six milk-fluoridation schemes operating in Thailand.
  It was also necessary to inspect the dairies once a year. This was done by three dental officers (@ THB 230.77) and took 16 h per dairy.
3. Promotion and reports: a dental nurse (@ THB 173/per hour) made a 3-h visit to each of the 435 BMA schools in Bangkok once a year to answer questions, promote and explain the program. As part of this visit, the nurse collected signed consent forms from parents of children entering the fluoridated-milk program. Consent forms were printed at a cost of THB 0.5 per form.

A promotional booklet was prepared every 2 years at a cost of THB 100,000. This cost was shared by the six schemes operating in Thailand (i.e., THB 16,667 per plant).

6) Follow-up teacher meeting: at the commencement of each provincial milk-fluoridation scheme a launch meeting was held to provide information about the scheme to all involved parties. The participants are the responsible teachers or school principals of all schools, local authorities, dentists and dairy technicians. To ensure the sustainability of the scheme, every 3 years after this a follow-up teacher meeting was organized to inform and provide updates on the progression of the scheme. In the case of Bangkok, these consisted of 435 BMA Teachers (@ THB 30.000/month); 65 BMA Health and Education Authorities (@ THB 30.000/month); 60 Dentists (@ THB 40.000/month); and 40 Dairy technicians (@ THB 30.000/month), in total 600. The cost for each meeting was, according to information from the DOH, THB 300,000 (THB 500/person: including lunch and meeting facilities).
7) Treatment costs: restoration and extraction costs were based on public sector fees (THB 170). No difference was made by the number of tooth surfaces affected. Both procedures include the costs of the dental examinations. It was assumed that:

- Increases in the decayed and missing components of the DMFS occurred at the same rate in each year of the study.
- The treatment costs associated with increases in the decayed and missing components of the DMFS score occurred in the year of the increment.
- All decayed tooth surfaces would be treated.
- One dental session would be necessary to fill a decayed tooth.
- There would be no need to replace restorations.

8) Opportunity costs: to calculate family resources used in dental treatments (i.e., transport costs and productivity losses), we assumed that the duration of a dental visit would be 4 h. This time included public transportation to and from the local health centre and the dental treatment.

- Work productivity losses were calculated for the adult accompanying the child by multiplying the number of dental visits required for each group by the minimum hourly salary in 2011. Costs of travelling time plus treatment time were estimated. Costs of work productivity losses were calculated at the value of the minimum monthly salary in 2011 (Minimum Salary THB 37.5 per hour). Time loss for children was not included.
- Transportation costs were calculated using public transportation in Bangkok (THB 16 per adult person, THB 8 per child per trip).

Cost categories for the control community:

1. Treatment costs.
2. Cost of travel to health centre. Cost estimates for child and parent travel.
3. Cost of productivity losses were calculated as above.

### Discounting

All costs were discounted retrospectively. Data on the total cost of implementing the fluoridated-milk program in the 2011 financial year (Year 5) was requested from the different sources and deflated to 2005 (Year 0) at 3.25% per year [19]. The assumption was that the annual cost of the program remained constant over time. Health benefits (i.e., dental caries) were not discounted [20].

### Outcome measures

The primary outcome was the number of permanent tooth surfaces with active dental caries, the number of restored surfaces due to dental caries and the number of missing permanent teeth due to dental caries, as measured by the DMFS index. For the assessment of the effectiveness of the intervention, data from two schools were used: Saunbua, one school out of 13 schools in a district was chosen to be the intervention group (fluoridated milk drinking group) (*n* = 112) and Payatai, a comparable school in a nearby district that received school milk without added fluoride was chosen as the comparison group (*n* = 134) [21]. Using the classroom as the sampling unit, the sample was selected by simple random. The same children, in both the control and test schools, were examined each year for 6 years.

Clinical examinations were conducted by six trained and calibrated examiners (Kappa statistics > 0.80) following WHO criteria and recommendations for oral health data collection [22], using dental mirrors, the WHO CPI probe, and a mobile dental unit with artificial light. Calibration exercises were conducted annually before data collection. At baseline (2000) the two communities were equivalent in terms of dental caries history and calculations were corrected for baseline caries. Data were also collected for dental caries risks including parent socioeconomic status, sugar consumption, oral health behaviour (i.e., tooth brushing) and fluoride use history [21]. Data analysis showed no statistical differences between the experimental and control groups in terms of risks factors. A report on this detail is being prepared and will be published under a separate cover.

The outcome after 6 years indicated that the overall caries experience (DMFS) of children aged 6 to 12-years from Suanbua was 1.06. On the other hand, among children living in Payatai, the non-fluoridated community, the mean DMFS index was 1.60 for the same period. The difference of 0.54 DMFS in 2005 between the two communities, which represents a reduction of 34%, was statistically significant (*p* < 0.05) [6].

### Sensitivity analysis

For the sensitivity analysis, the following assumptions were varied one at a time (i.e., one-way sensitivity analysis):

- DMFS outcome at the high and low boundaries of caries reduction, as reported in the literature (i.e., 31% – 58%) [23], and
- Social discounting varied from 0% and 5% [11].

## Results

Calculations based on data provided from the Royal Chitralada Project indicate that the undiscounted investment necessary to produce both Pasteurized fluoridated-milk and UHT fluoridated-milk is THB(2011) 386,019.00 (See Table 1).

**Table 1:** Summary of annual total costs (by cost category) associated with the milk-fluoridation program in Bangkok, Thailand, 2011

Cost category	Pasteurized milk THB^a^(2011)	UHT milk THB^a^(2011)
Investments costs:		
Production equipment		
Dosing pump equipment (55,000.00 × 5 diaries)	275,000	66,549
Laboratory equipment	44,470	
Total Investment	319,470	66,549
Total Investment costs		386,019
Production costs:		
Cost of analysis (reactives and calibration) per year	43,168	13,942
Production Cost (75,000 units/day) per year	40,236	16,430
Subtotal per year	83,404	30,488
Total cost production per year		113,892
Quality control costs:		
Cost transport samples a year (THB 200 per trip)		1600
Cost of samples analysis a year (THB 30 per samples)		8640
Personnel costs (THB 138.46/h; 66 h per year)		9138
Urinary Analysis cost a year (32,800/6 diaries)		5467
Total quality control per year		24,845
Promotion, reports and management costs:		
Program Coordinator		48,000
Administrative assistant		28,800
Office rent and services		9720
Dental nurse 3 h/year per school (*n* = 435)		225,865
Consent form printing (THB 0.5 each. First year only)		37,500
Teacher meetings salary (At commencement and every 3 years)		858,462
Teacher meetings organisation (At commencement and every 3 years)		300,000
Report to Princess		1667
Booklet for promotion scheme (Every 2 years)		33,334
Inspection diaries (3 dental officers @ THB 231/h; 16 h × 6 diaries)		66,462
Total promotion, reports and management costs per year		1,609,810

^a^Thailand Baht (THB) [1 US$ = TBH.(2011) 30.00]

Production costs. The daily milk consumption in children is 200 ml of pasteurized milk (bag) per day during school days and one carton per day of UHT milk during holidays. Producing 75,000 bags for 200 school days (@ THB 0.00556 per bag) would cost THB 43,168 per year. To produce 75,000 cartons for 60 vacation days (@ THB 0.00678 per carton) would cost THB 16,430 per year. Thus, the total undiscounted annual cost to add fluoride to milk distributed by the BMA would be THB 113,892 per year.

Milk quality control analysis and monitoring. To check for the consistent provision of appropriate concentrations of NaF, milk samples are delivered from schools to the Ministry of Health, at a cost of THB 1600 a year (THB 200 per trip × 8 times a year). There is a cost of THB 30 per sample for the fluoride analysis at the Ministry of Health. Thus, for six samples per dairy, eight times a year, the cost would be THB 8640.

The laboratory technician’s costs to conduct the analyses at the Ministry of Health would be THB 9138 (THB 138.46/h; 66 h per year). The cost of the urinary analysis would be THB 5467.

Promotion, reports, and management. Assuming a wage of THB 40,000 a month for a fulltime equivalent position, the coordinator’s salary would be THB 48,000 a year. The coordinator AA’s salary would be THB 28,800 a year.

The office space rent would be THB 8100 a year (THB 39 per hour, 4 h a week, 52 weeks a year). Additionally, it is estimated that services including electricity, water, internet and phone would cost THB 1620.

A dental nurse would make 3 h visits to each school once a year to answer questions, promote and explain the program in 435 BMA schools. In order to visit all the schools the dental nurse would have to work 1305 h (@THB 173 per hour), equivalent to THB 225,865 a year.

Consent forms (@THB 0.5 per form) were printed in year 1 only. Assuming 75,000 children would need a form filled-in and signed by their parents or guardians, the cost would be THB 37,500.

Salaries for 600 professionals participating in an 8-h teacher meeting would be THB 858,462. The cost of organising the teachers’ meeting was THB 300,000. This meeting took place at commencement and in Year 4 of the scheme.

The report and presentation to the Princess every year had a cost of THB 1667 a year. The cost of the promotion booklet, to be prepared every 2 years, would be THB 33,334.

The once-a-year inspection of the dairies was done by three dental officers who take 16 h per dairy, at a salary of THB 230.77 per hour, per person. The total cost of inspecting the 5 dairies for 16 h a year each was THB 66,462. Thus, the expected undiscounted annual cost of Promotion, Reports and Management would be THB 1,609,810.

Cost of the Program (See Table 2). Assuming that the annual cost of the fluoridated milk scheme remained constant over time, the value for 2011 was applied retrospectively to all previous years and deflated at 3.25% per year. The total discounted cost of the program offered to 75,000 children for 6 years was THB 5,345,048. That is, the cost was THB 71.26 per child for a six-year program, or THB 11.88 per child per year. This cost included the fluoridation of the milk, tests, transportation, promotion and administration.

**Table 2:** Summary of total discounted costs over six-years associated with the milk-fluoridation program and proportion of each category of the total. Bangkok, Thailand, 2011

Present cost of program for 6 years^b^	Annual discounted cost THB^a^ (2011)	% of total cost
Initial Investments	311,903	7.16
Production costs of fluoridated milk	629,857	14.33
Milk quality control analysis and monitoring	137,470	3.13
Promotion, Reports, and Management	4,265,818	75.38
Total present costs	5,345,048	100.00

^a^Thailand Baht (THB) [1 US$ = TBH.(2011) 30.00]; ^b^Discount rate = 3.25%

The largest cost component was promotion, reports, and management: 75.38% of the total annual fluoridated-milk discounted cost. Production costs and milk quality control analysis and monitoring costs accounted for 141.33% and 3.13%, respectively. Initial investment accounted for 7.16% of the total discounted cost.

The estimated discounted cost of dental treatment over the 6 years for the intervention and control groups is shown in Table 3. Costs were about 33.5% higher in the control group (THB 40,549,920 or THB 90.1 per child per year) compared with the intervention group (THB 26,927,693 or THB 59.8 per child per year).

**Table 3:** Summary of costs of discounted dental treatments in the intervention and control communities after six-years of program in Bangkok, Thailand, 2011

Treatment item	Total test community (THB)^a^	Total control community (THB)
Restorations	12,410,149	21,817,620
Opportunity costs (travel productivity losses)	14,517,544	18,732,300
Total present costs^b^	26,927,693	40,549,920

^a^Thailand Baht (THB) [1 US$ = TBH.(2011) 30.00]; ^b^Discount rate = 3.25%

When the costs of operating the program and the costs of dental treatment in the test community were compared with the costs in the non-fluoridated community, it was found that a public investment of THB 5,345,048 (or THB 11.88 per child per year) over 6 years resulted in a saving of THB 8,177,179 in societal costs attributable to the preventive program over the 6 years. This investment would result in a reduction of 40,500 DMFS avoided within a community with a childhood population of 75.000 [DMFS avoided: 75,000 x (− 0.54)] (See Table 4). Thus, investing in the program would not only result in a reduction in disease, but in a net financial saving to the community.

**Table 4:** Total costs and benefits for the overall milk-fluoridation program in Bangkok, Thailand, 2011

		THB^a^ (2011)
A	Total cost test community	32,372,741
B	Total cost control community	40,549,920
C	Incremental cost (or saving)	(8,177,179)
D	Incremental benefits (DMFS avoided; 75,000 x (− 0.54))	40,500

^a^Thailand Baht (THB) [1 US$ = TBH.(2011) 30.00]

Sensitivity analysis would result in net savings ranging from THB 18,597,122 to THB 7,920,711 after 6 years for the intervention group, when compared to the non-fluoridated group (See Table 5). This range of variability was caused by uncertainties of the effectiveness of the milk-fluoridation scheme. As expected, the most favourable result was gained by using the best-case scenario of the effectiveness assumption, that is, 58% caries reduction. Conversely, the least favourable result was found using the worst-case scenario of effectiveness (i.e., 31%).

**Table 5:** Sensitivity analysis milk-fluoridation program in Bangkok, Thailand, 2011

Variation of assumption	Program costs THB^a^	Cost treatment F-community THB^a^	Cost treatment control community THB^a^	Incremental costs THB^a^	Incremental benefits THB^a^
Primary analysis	5,345,048	26,927,693	40,549,920	8,177,179	40,500
Effectiveness					
Best case scenario (58%)	5,345,048	16,607,750	40,549,920	18,597,122	69,600
Worst case scenario (31%)	5,345,048	27,284,161	40,549,920	7,920,711	37,200
Discount rate					
0%	5,923,610	29,256,000	44,160,000	8,980,390	40,500
5%	5,079,608	25,833,838	38,994,474	8,081,028	40,500

^a^Thailand Baht (THB) 2011 [1 US$ = TBH.(2011) 30.00]

## Discussion

Our primary EE estimated that if a dental caries prevention program using fluoridated-milk from the BMA were available for 75,000 children aged from 6 to 12 years, the net saving from a societal perspective in dental treatment would total THB 8,177,179 over 6 years. These societal savings would be achieved at a yearly cost to a government-sponsored agency of THB 71.26 per child for a 6 year program, or THB 11.88 per child per year. That is, spending THB 11.88 per year per child would save THB 18.17 per child per year, for a 6 year program. From the family perspective, the net present value of savings, due to savings in production losses, transportation, and other uses of time, would be THB 2.55 {([40,549,920–26,927,693]/75,000)/71.26} for each THB spent on the milk-fluoridation program.

Assumptions and uncertainties were explored in the sensitivity analysis to test the robustness of the results. The milk-fluoridation alternative remained dominant even when more stringent and pessimistic assumptions were evaluated. The sensitivity analysis yielded confidence intervals (THB 18,597,122 to THB 7,920,711) resulting from the ranges of variation of two key input parameters. Thus, even with a lower confidence limit in dental caries reduction, the milk-fluoridation program would still be a cost-effective health intervention compared to current oral health care.

Compared with other milk-fluoridation programs [9, 10], the present study appears to be less cost-effective, but still represents an efficient use of resources. This somewhat lower range was highly influenced by the fixed administrative costs included in the total costs. In particular, the cost of promotion and teacher meetings accounted for 79.81% of the promotion, reports and management costs category, over the total period of the scheme. Additionally, an environment of low caries prevalence (i.e., DMFS 1.1 to 1.6) [24] limited the cost-effectiveness of the fluorides program [25]. An EE in areas where communities are at higher risk of dental caries would provide a more cost-effective result.

The results of this research are presented in incremental costs and incremental benefits (i.e., THB 8,177,179 of cost-savings and 40,500 DMFS avoided). However, there are many ways in which they do not account for all possible costs and benefits of a program. Thus, this analysis can be considered conservative. The use of a number of assumptions might have underestimated the savings of the program [26]. For example, only the costs of the initial treatment were considered. Additional costs due to potentially costlier treatments (e.g., cost of space retainers after early extraction of deciduous teeth, or pulpal therapies) were not considered. On the other hand, the study assumed that the decay component of the DMFS index was restored in the year of the increment. There is no guarantee that the necessary treatment would be undertaken.

Pain, infection and tooth loss are the most common consequences of oral disease. The contribution of a healthy dentition to quality of life was not quantified, but is probably highly valued by most people [25]. Moreover, there are many non-health benefits of a preventive approach which were not considered. This analysis ignores issues around social justice and a decrease of inequalities in health. These issues are complex and difficult to assess within the constraints of a research framework, but are not outside the realm of EE [26]. Their inclusion would provide a closer picture of the advantages in any preventive program.

The current study represents only the first step in determining whether a specific oral health care program is an efficient use of scarce community resources. Although appropriate data sources were available for most assumptions used, follow-up studies are required to avoid assumptions. Thus, future studies need to be done under more generalizable conditions and it would also be appropriate to prospectively collect information regarding actual treatment costs along with those of the intervention. Such an economic evaluation would decrease the need to undertake sensitivity analyses [11].

## Conclusions

The purpose of this evaluation was to assess the cost-effectiveness, from a societal viewpoint, of a dental caries prevention program using fluoridated milk in the city of Bangkok, Thailand, for children from 6 to 12 years old, versus non-intervention. While the analysis has inherent limitations due to its dependence on a range of assumptions, the results provide important information for further economic evaluation, and may help local program managers to determine the average cost per child and per DMFS avoided of providing fluoridate milk, under the conditions prevailing in Thailand. Since the analysis indicated consistent cost-savings and health gains, in terms of dental caries averted, a reasonable conclusion is that the economic and human benefits to society are significant.

## Abbreviations

AA: Administrative assistant
BMA: Bangkok Metropolitan Administration
CEA: Cost-effectiveness analysis
DMFS: The DMFS index represents the arithmetic average number of tooth surfaces (S) that are decayed (D), missing (M), and filled (F) as a result of caries occurring in permanent dentition
DOH: Department of Health
EE: Economic evaluations
NaF: Sodium fluoride
THB: Thailand Baht
UHT: Ultra-high temperature processing

## Footnotes

1 The Royal Chitralada Projects are projects based in Chitralada Villa, the royal residence of their Majesties the King and Queen in Bangkok, conducted to tests and experiments in agriculture and agricultural products. The Chitralada Villa include rice fields, fish ponds, a dairy farm, tissue culture plant and various kinds of pilot factories. Wattana T. The Royal Chitralada Projects initiated by H.M. King Bhumibol. 2002. http://www.thaiwaysmagazine.com/thai_article/30-07_the_royal_chitralada/the_royal_chitralada.html. (accessed 14.10.17)
